# Anxiety Symptoms and Comorbid Anxiety Disorders in Adolescents with Anorexia Nervosa: A Scoping Review and Proposal for a Management Algorithm

**DOI:** 10.3390/nu18213551

**Published:** 2026-10-28

**Authors:** Aline Chandran, France Hirot, Anaël Ayrolles, Nathalie Godart

**Affiliations:** 1 Fondation de Santé des Étudiants de France, 75014 Paris, France; france.hirot@fsef.net; 2 CESP, INSERM, UMR 1018, Université Paris-Sud, Université Versailles Saint Quentin, Université Paris-Saclay, 94800 Villejuif, France; 3 Department of Child and Adolescent Psychiatry, Robert Debré University Hospital, AP-HP.Nord, Université Paris Cité, 75019 Paris, France; anael.ayrolles@aphp.fr; 4 Human Genetics and Cognitive Functions, Institut Pasteur, 75015 Paris, France; 5 UFR Simone Veil, Université Versailles Saint Quentin, 78180 Montigny-Le-Bretonneux, France

**Keywords:** anorexia nervosa, adolescents, anxiety symptoms, anxiety disorders, treatments

## Abstract

Background: Anorexia nervosa (AN) frequently begins in adolescence and is commonly associated with anxiety symptoms. Their management remains insufficiently established: the overlap between the symptoms of AN, undernutrition and anxiety disorders makes comorbid disorders difficult to diagnose, and no management strategy has been formalized. This work aims to map how international clinical practice guidelines and the recent literature address anxiety symptoms and comorbid anxiety disorders in adolescents with AN, and to propose a management algorithm on that basis. Methods: A total of 11 clinical practice guidelines on AN published in French or English were identified, two of them specific to children and adolescents, and were supplemented by a PubMed and Embase search covering January 2023 to January 2026 (n = 18 publications) following the PRISMA methodology for scoping review. Results: The guidelines consistently recommend screening for comorbid anxiety disorders, while acknowledging how difficult it is to disentangle symptoms due to undernutrition, to AN itself, or to a comorbid disorder. They recommend considering nutritional status and the course of anxiety symptoms during weight restoration before introducing any psychotropic medication, and treating comorbid disorders according to their severity and to the guidelines specific to each. No trial has evaluated the treatment of a comorbid anxiety disorder in adolescents with AN. Conclusions: Based on this review and the guidelines on anxiety disorders, we designed a therapeutic algorithm for isolated anxiety symptoms, uncertain situations, and established comorbid diagnoses, indicating the basis of each step.

## 1. Introduction

Anorexia nervosa frequently emerges during adolescence: its peak age at onset is 15.5 years and its median age at onset 17 years, with 55% of cases beginning before the age of 18 and 78% before 25 [1]. Among adolescents aged 12 to 17 years, it affects 0.5% of girls and 0.03% of boys [2]. More than 25% of cases follow a chronic course, with elevated morbidity and mortality [3]. It is therefore a major public health concern, since eating disorders in adolescence compromise adult outcomes in terms of physical health, mental health and social integration [4].

AN is also associated with numerous comorbidities [5], which may occur before, during or after the eating disorder (ED) [6]. Anxiety and mood disorders are the most commonly reported comorbidities in this population [7,8]. Anxiety and AN are closely intertwined, and their developmental trajectories influence one another [9]. AN is more frequent among adolescents and young adults with a diagnosis of anxiety disorder [10]. The disorders most often reported are social anxiety disorder, panic disorder, agoraphobia, generalized anxiety disorder (GAD) and separation anxiety disorder [11] along with obsessive–compulsive disorder (OCD) [12]. The relationship between AN and anxiety is bidirectional. Anxiety may also worsen the prognosis of the ED [13], and untreated comorbidities can prevent remission of AN [14].

Identifying such comorbidities remains difficult, because the symptoms of AN and those of anxiety disorders largely overlap. Undernutrition itself can induce anxiety [15], and refeeding is also highly anxiety-inducing [16], confronting patients with situations that are difficult to experience. Distinguishing isolated anxiety symptoms related to undernutrition or to anorexia nervosa from those inherent to a comorbid anxiety disorder is a genuine challenge. Establishing or ruling out this diagnosis determines the therapeutic approach [15].

Diagnosis and treatment strategies remain insufficiently defined. The literature recommends treating comorbidities, such as anxiety disorders or OCD, when they persist after nutritional status improves [14]. As for depression in AN [17], no management strategy has been specifically formalized. Meanwhile, clinicians in specialist ED department frequently prescribe psychotropic drugs off-label in AN [17,18]. Previous reviews have addressed the epidemiological association between anxiety and eating disorders [9,13] or the pharmacological treatment of anxiety disorders or eating disorders [13,19]. To our knowledge, none has specifically addressed the differentiation between anxiety symptoms related to AN or undernutrition and those reflecting a comorbid anxiety disorder, nor the therapeutic approaches that should be adopted in these different clinical situations.

To bridge this gap between practice and evidence, we conducted a scoping review to map how international guidelines and the recent literature address anxiety symptoms and comorbid anxiety disorders and their treatment in anorexia nervosa. Based on this review and the guidelines on anxiety disorders, we designed a therapeutic algorithm for clinical management of these situations.

## 2. Materials and Methods

We conducted a scoping review following the Preferred Reporting Items for Systematic reviews and Meta-Analyses extension for Scoping Reviews (PRISMA-ScR) methodology [20]. Two sources were combined: all international guidelines on AN published in French or English, and more recently published studies retrieved from PubMed and Embase databases.

### 2.1. Eligibility Criteria

We included comprehensive clinical practice guidelines on AN published in French or English by the government agency or learned societies responsible in each country.

When the guidelines had been revised, only the most recent version was used. We then searched for studies on the management of anxiety in AN published from 2023 onwards (to assess the most recent literature). We applied a hierarchy of evidence: we retained the highest degree of evidence available according to the classic hierarchical pyramid [21]: meta-analyses where they existed, otherwise systematic reviews, then randomized trials or trials not covered by those reviews, and finally, where no other source existed, open-label studies. This was a source-selection rule, intended to summarize the available evidence studies, as previously done in this field [18]; it does not constitute a formal appraisal of the methodological quality or certainty of the evidence, which was not performed, in line with the scoping-review methodology [21]. Case studies were excluded (see Figure 1).

We defined adolescence as the period from 10 to 24 years, following Sawyer et al. [22], who propose this range on the basis of the earlier onset of puberty and the later timing of adult role transitions, and which corresponds to the population seen in specialized eating-disorder services. Studies were eligible if they included participants with AN, alone or within transdiagnostic eating-disorder samples, and reported an anxiety-related outcome (anxiety symptoms, anxiety disorders or obsessive-compulsive symptoms); reviews covering several eating disorders were retained when they reported results for AN. Studies conducted only in other eating disorders or in other psychiatric conditions were excluded. Because evidence specific to adolescents with AN is scarce, we also included studies conducted in adults and in mixed-age samples: for most interventions, these constitute the only available data on anxiety outcomes in AN. They provide indirect evidence, and the population of each study is stated throughout the Results and in Table S2.

### 2.2. Literature Search Strategy

From the guidelines on AN, we extracted all content relating to anxiety symptoms and anxiety disorders. Guidelines were identified through a structured search conducted on 15 October 2026, following the documentary search principles of the French National Authority for Health [23]. We searched (i) PubMed, using the equation given in Table S3, and (ii) the websites of the national health agencies and learned societies responsible for guidelines in psychiatry, child and adolescent psychiatry, pediatrics and eating disorders (listed in Table S3). Reference lists of included guidelines and of two published syntheses of eating-disorder guidelines [24,25] were hand-searched. The authors’ initial identification, including a search with a generative AI tool (Copilot version 2.20261026.24.0), was used only as a completeness check, as this step is not reproducible. No date restriction was applied. Inclusion criteria were: (a) comprehensive guidelines on the specialist management of eating disorders including AN, developed by or for psychiatric or multidisciplinary eating-disorder services, consistent with the population targeted by this review; (b) full text available in French or English; (c) issued by a government agency or a national or international learned society; (d) most recent version when a guideline had been revised. Guidelines intended exclusively for primary care or community pediatric practice, which address recognition, initial assessment and referral, were excluded, as were guidelines restricted to a single aspect of care (screening, dietetic, somatic or emergency management, or transitions of care) and consensus documents without institutional endorsement, with the exception of the WFSBP guidelines, which address pharmacological treatment, the central topic of this review. The documents identified, excluded (with reasons) and included are reported in Table S3.

From the databased, we selected articles published between January 2023 and January 2026 (the last search was conducted on 4 January 2026), using the following equations:

- Search Equations in PubMed:

○ (Anorexia nervosa) AND (treatment or pharmacotherapy or psychotropic drug or pharmacology or family therapy or exercise or physical activity) AND (anxiety or anxiety disorder or anxiety symptom)
○ “Anorexia Nervosa/therapy”[Mesh] AND (“Psychotropic Drugs”[Mesh] OR “psychotropic drug*” OR “Psychopharmacology”[Mesh] OR “psychopharmacology” OR “Psychotherapy”[Mesh] OR “psychotherapy” OR “Family Therapy”[Mesh] OR “family therapy” OR “exercise*”) AND (“Anxiety”[Mesh] OR “Anxiety” OR “Anxiety Disorders”[Mesh] OR “Anxiety Disorder*”)

- Search Equation in Embase:

○ ‘anorexia nervosa’/exp/dm_th AND (‘psychotropic agent’/exp OR ‘psychotropic drug*’ OR ‘psychopharmacology’/exp OR ‘psychopharmacology’ OR ‘psychotherapy’/exp OR ‘psychotherapy’ OR ‘family therapy’/exp OR ‘family therapy’ OR ‘exercise*’) AND (‘anxiety’/exp OR ‘anxiety’ OR ‘anxiety disorder’/exp OR ‘anxiety disorder*’) AND [2023 to 2026]/pd

### 2.3. Selection Process

Screening and data extraction were performed independently and in duplicate by two reviewers (AC, NG). Discrepancies were discussed between the two reviewers.

Both reviewers extracted the data in duplicate. We synthesized the material narratively and grouped it under the recurrent clinical themes that emerged from the documents. Themes covered identification and treatment: taking undernutrition into account, when and how to treat anxiety comorbidities, psychotherapies, pharmacological treatments, comorbid anxiety disorders, hospitalization, specific features of the treatment of anxiety disorders in children and adolescents, and other approaches and future prospects for the treatment of anxiety comorbidities and symptoms in AN.

### 2.4. Design of a Therapeutic Algorithm for Anxiety Manifestations in Adolescents with AN

Following this literature review, and given that the available evidence did not allow the formulation of clear recommendations for the management of anxiety symptoms in anorexia nervosa, we propose a clinical algorithm, detailed in the Section 4.2.

The algorithm was developed within a clinical framework based on a psychiatric assessment aimed at identifying the anxiety symptoms and signs present and, when appropriate, detecting comorbid conditions. This approach is consistent with international clinical recommendations. For example the APA guidelines mentioned “The relationship between eating disorders and psychiatric symptoms is complex; careful clinical assessment is needed to discern whether symptoms of depression, anxiety, or obsessiveness reflect an independent co-occurring disorder or have developed as a result of the eating disorder” [16].

A preliminary clinical assessment allowed the identification of three clinical situations: isolated anxiety symptoms, a confirmed comorbid anxiety disorder, and anxiety symptoms of uncertain origin. The findings of the scoping review were grouped under these three clinical categories into a hierarchical framework of first-line and second-line management strategies. Where guidelines diverged, the more cautious recommendation was retained. When data was not available, we referred to the guidelines for child and adolescent anxiety disorders [26,27,28,29,30,31,32], available evidence regarding pharmacological treatments in eating disorders [33,34], the authors’ clinical experience and the clinical experience reported in the literature. The resulting draft was reviewed by all authors, and disagreements were resolved by discussion until consensus.

## 3. Results

We identified 11 clinical practice guidelines on AN: Spanish (2009) [35], French (2010 and 2022) [36,37,38], Australian and New Zealand (2014) [39], German (2018) [40], British (2017, revised 2020) [41], WFSBP guideline (revised 2023) [42], Americans (2015, 2023) [16,43], Scottish (2022) [44], Brazilian (2026) [45] (see Supplementary Materials: Table S1). Two of them is specific to children and adolescents, the French 2022 guideline on early-onset anorexia nervosa [38] and the AACAP practice parameter [43]. Overall, the guidelines are largely consensual, although some topics are addressed in certain guidelines and not in others. Guidelines did not identify any high-or moderate- evidence supporting treatment strategies for anxiety symptoms or comorbidity in anorexia nervosa.

The complementary search added 18 more recent publications: five systematic reviews with meta-analysis, three systematic reviews and one scoping review without meta-analysis, four narrative reviews, two randomized trials and three other primary studies. Findings obtained specifically in adolescents are identified as such throughout this section. Where no such indication is given, the data come from adult or mixed samples. As only two of the retrieved publications reported anxiety outcomes specifically in adolescents [46,47]), we included work carried out in samples comprising adults.

### 3.1. Screening

The guidelines agree both on the need to identify comorbidities in AN, but also on the difficulty of doing so. Deciding whether one or several diagnoses apply is often difficult [35,37]. Anxiety symptoms arising from AN itself linked to body shape perception, undernutrition, nutritional rehabilitation, compensatory behaviors, or personality traits [16,35,39] closely resemble those of a comorbid anxiety disorders. Moreover, undernutrition and compensatory behaviors can themselves produce anxiety and obsessive-compulsive symptoms [35,39]. Anxiety symptoms or irritability may resurge during remission, as weight is regained and as the patients approach a weight they dread. These symptoms subside gradually over the months that follow weight restoration, provided that the weight is maintained and that restrictive, compulsive or purging behaviors do not return. If, by contrast, obsessive thoughts and compulsions unrelated to food persist, this suggests the presence of co-occurring disorders and raises the question of their respective treatment [16,43]. The link between eating disorders and psychiatric symptoms is complex: every form of anxiety disorder (social phobia, GAD) and OCD may occur during AN [39]. Only a thorough clinical assessment can establish whether the symptoms observed reflect a comorbid psychiatric disorder or are manifestations of AN [16].

### 3.2. Treatment

#### 3.2.1. Taking Undernutrition into Account

Guidelines advise delaying medication for comorbid disorders, particularly anxiety disorders, until nutritional rehabilitation has progressed far enough to show whether the anxiety symptoms were due to undernutrition alone [39,43]. Similarly, obsessive symptoms are also frequently caused or increased by low body weight and may improve with weight restoration without any additional specific treatment [35,40]. Treating the eating disorder can therefore sometimes contribute to treating the comorbid symptoms [35,41], and the recent literature confirms that nutritional rehabilitation reduces anxiety symptoms in AN in an observational study including 50 adolescents and young adults (aged 11–26 years) [48]. When a psychotropic drug is prescribed in AN for associated comorbidity, undernutrition and compensatory behaviors may alter both its efficacy and its side-effect risks [41]. Caution is required with all psychotropic drugs, because the physical complications of AN may place individuals at higher risk of adverse effects [39,44]. QT-prolonging drugs, alone or in combination with other drugs, require particular caution in patients with purging behaviors [16].

#### 3.2.2. When and How Should Anxiety Comorbidities Be Treated?

Despite these cautions, the guidelines emphasize the importance of treating comorbid psychiatric disorders, even though there is little evidence regarding the efficacy of treatments in these situations [41]. Severity may occasionally require treating the comorbidity before the eating disorder [41]. The choice between simultaneous and sequential treatments should take into account the severity and complexity of both conditions, the patient’s level of functioning, and the preferences of the patient as well as, where appropriate, of their relatives [41]. Comorbidities should otherwise be treated as their own guidelines propose [41,43].

#### 3.2.3. Psychotherapy

Psychotherapy, and cognitive behavioral therapy (CBT) in particular, is the first-line treatment for comorbid anxiety disorders ahead of pharmacotherapy [36,38]. The approach should follow the guidelines for the comorbid disorder concerned [40], and medication is recommended as a second-line option, after psychotherapy and in combination with it [39]. A recent meta-analysis found various forms of CBT effective on anxiety symptoms in AN (CBT combined with nutritional counseling, group CBT, online CBT) [49]. The effect size was medium (see Table S2), but small samples were used, with three studies comprising a total of 58 participants (adolescents and adults) [49]. A randomized trial also showed moderate to large improvements in anxiety symptoms after PAT-AN (Positive Affect Treatment for Anorexia Nervosa) delivered to 20 adults with AN, maintained three months after therapy ended [50].

#### 3.2.4. Pharmacological Treatments

- Antidepressants

Antidepressants are consensually recommended for anxiety disorders and OCD co-occurring with AN when these do not improve with weight restoration [35,37,40], always taking the benefit–risk balance into account [16,35]. The guidelines diverge on certain specific points. French guidelines and the APA recommend the use of antidepressants to treat anxiety symptoms in persons with AN [16,37], whereas the Australian guidelines highlight the limited evidence supporting the use of antidepressants in ED [39]. The French guidelines advise using tricyclic antidepressants with caution in undernourished patients because of their adverse effects (QT prolongation) [37], whereas the German guidelines note a theoretical value of clomipramine (a tricyclic antidepressant) for the treatment of anorexia nervosa given its use in the treatment of OCD [40]. The pediatric guidelines are more restrictive than those developed in adults. They state that SSRIs are not effective in severely undernourished children and should be introduced only once sufficient weight has been regained, given that the risk of iatrogenic effects is correlated with the degree of undernutrition [36].

A recent systematic review of 51 studies on the pharmacological treatment of anxiety symptoms in eating disorders identified eight studies on antidepressants, comprising a total of 213 adults. This review found fluoxetine (five studies) and citalopram (one study) to be effective, with a limited level of evidence for the efficacy of citalopram (limited recommendation for its use) on anxiety symptoms in AN [13]. Consistently, a meta-analysis concluded that fluoxetine significantly improves obsessive–compulsive symptomatology compared with placebo in AN (meta-analysis of three randomized controlled trials encompassing 159 adult women) [19].

- Antipsychotics

Antipsychotics may be considered in eating disorders for their effect on reducing anxiety or rumination [35,39], with caution because of QT prolongation [16,35,37]. Antipsychotics reduce stress, the often marked urge to move, and circular thinking about eating disorder-related themes such as food, body shape or weight [40]. Four principles are mentioned for the administration of antipsychotics in AN:

- The required dose is generally low.
- The indication derives from the comorbid symptoms associated with AN, and the treatment duration depends on the course of those symptoms, not on the eating disorder diagnosis.
- Antipsychotics with the fewest extrapyramidal adverse effects are preferred.
- Adherence is crucial, and the benefit of the drug must be explained to the patient [40].

Low doses of olanzapine are particularly useful in highly anxious patients with obsessive ruminations related to food [35,39]. Treatment is started at a low dose (2.5 mg) and increased if necessary [40]. Olanzapine is reported to reduce obsessive symptoms, compulsions and circular thinking [40]. However, its adverse effects (glucose dysregulation, metabolic syndrome, akathisia, and extrapyramidal effects) must be taken into consideration [16,45].

Recent reviews reporting two open-label studies supported a limited level of evidence for the efficacy of olanzapine on anxiety in 22 adolescents and adults with AN [51], but this efficacy has not been significantly confirmed in the three randomized placebo-controlled trials in 209 adult participants pooled in a meta-analysis [19].

- Anxiolytics

Benzodiazepines have not been shown to be effective as anxiolytics and carry a risk of misuse [16,40]; their high potential for dependence and their negative effects on learning markedly limit their use [40]. Only one randomized clinical trial is available, conducted on 20 participants with a mean age of 26 years with AN, and it did not show any efficacy of alprazolam on pre-meal anxiety symptoms [13,14].

#### 3.2.5. Treatment of Comorbid Anxiety Disorders

We identified no study evaluating the treatment of a comorbid anxiety disorder, as opposed to anxiety symptoms, in patients with AN.

#### 3.2.6. Hospitalization

The French guidelines indicate that the intensity of comorbidities (including anxiety disorders or OCD) may indicate hospitalization [37].

#### 3.2.7. Specific Features of the Treatment of Anxiety Disorders in Children and Adolescents

The American guidelines support antidepressants for co-occurring disorders in adolescents and young adults according to the benefit–risk balance [16]. In Australia and New Zealand, caution is recommended when using antidepressants and anxiolytics to relieve symptoms, with the evidence being considered insufficient in adolescents with AN [39]. The German and French guidelines caution against prescribing antidepressants to children [38] and to undernourished adolescents [40].

#### 3.2.8. Other Approaches and Future Prospects for the Treatment of Anxiety Comorbidities and Symptoms in Anorexia Nervosa

Some approaches do not appear in the international guidelines but are the subject of preliminary work in the recent literature, with an overall low degree of evidence and none specifically targeting a comorbid anxiety disorder in adolescents.

- Psychotropic Drugs under Investigation

Several compounds are being explored as potential therapeutic options. The WFSBP issues a weak recommendation for this ketamine treatment combined with a ketogenic diet [42]. A systematic review identified five studies on psychedelics and cannabinoids, comprising a total of 29 adults. The three case series on ketamine (an N-methyl-D-aspartate [NMDA] receptor antagonist) provided preliminary evidence supporting its use for anxiety symptoms in anorexia nervosa [13]. Psilocybin (a psychedelic compound) is also supported by a low level of evidence (an open label feasibility study in 10 adults with AN [13,52]); it might reduce anxiety as well as food-related preoccupations and rituals, and several clinical studies are ongoing [52]. Metreleptin (three publications reporting five adult and adolescent cases) and cannabidiol (CBD, one ongoing study) [14] are also beginning to be investigated as possible therapeutic options for the future.

- Neurostimulation

Non-invasive brain stimulation techniques (repetitive transcranial magnetic stimulation, rTMS, and transcranial direct current stimulation, tDCS) appear to contribute to a reduction in anxiety symptomatology in AN in a meta-analysis including three studies and 57 adult participants [53]. Another meta-analysis including three studies and 82 adults confirmed that, rTMS decreased anxiety symptoms in anorexia nervosa [54]. A systematic review based on only two case studies and six adult participants reached similar conclusions [55]. These data are preliminary, come exclusively from adult samples, and do not allow for conclusions on the use of neurostimulation for anxiety in adolescents with AN.

- Nutritional Approaches and the Gut Microbiota

Omega-3 supplements as an adjunctive treatment in anorexia nervosa have not shown evidence of efficacy on anxiety symptoms in randomized controlled trials (meta-analysis with two studies including 27 adolescents and adults and a systematic review with one study including 24 adolescents) [46,56]. The gut microbiota is beginning to be investigated in research and could potentially, in the future, represent an avenue for addressing anxiety comorbidities in anorexia nervosa [57].

- Mind–Body and Complementary Approaches

Preliminary studies suggest that virtual reality approaches may reduce anxiety symptoms related to food intake or to body-related preoccupations in three studies with 147 adolescents and adults and in a randomized study with 145 adolescents and adults [47,58]. An uncontrolled pilot study including 13 female adolescents with anorexia nervosa suggests that art therapy could reduce state anxiety after a single session and trait anxiety after four to six sessions, in combination with multidisciplinary care [59]. In a randomized trial in 68 adolescents and adults with AN, floatation therapy (floatation-REST, Reduced Environmental Stimulation Therapy) was associated with a short-term reduction in anxiety symptoms after a single session, longer-term effects were not established [60].

## 4. Discussion

This scoping review of international guidelines and the recent literature converges on several points. Anxiety comorbidities must be identified and are difficult to distinguish from symptoms of undernutrition or of AN itself, and nutritional restoration should come before any psychotropic treatment. Psychotropic prescriptions, common in clinical practice, rest on a low level of evidence. No trial has specifically evaluated the treatment of a comorbid anxiety disorder in adolescents with AN, and no management strategy has ever been formalized.

The main difficulty identified is therefore diagnostic rather than therapeutic, since assessment determines the therapeutic strategy. The symptomatic overlap is considerable: undernutrition and compensatory behaviors can induce anxiety, rumination and obsessive symptoms [15,39] that weight restoration frequently resolves without specific treatment [40]. Three clinical features nevertheless help to guide the diagnosis: a personal and family history of anxiety disorders or OCD predating AN [61], persistence of symptoms after nutritional rehabilitation [16], and, above all, the nature of the obsessions and compulsions, which are ego-syntonic when belonging to AN and ego-dystonic when they reflect genuine comorbid OCD [62].

This diagnostic difficulty carries two opposing risks: delayed treatment of an established anxiety disorder, or over-diagnosis leading to unnecessary prescriptions in adolescents who are particularly vulnerable to adverse effects [16,39]. No study supports a strategy specific to anxiety disorders in adolescents with AN. Following the guidelines that recommend treating comorbidities according to their own reference frameworks [41,43,44], we propose a management strategy combining data specific to AN with the general framework of the guidelines on anxiety disorders in adolescents. We summarize that framework first, then propose an algorithm by clinical situation.

### 4.1. General Framework of the Guidelines on Anxiety Disorders in Adolescents

The guidelines for anxiety in adolescents are few, and the English NICE guidelines cover only social anxiety disorder [26]. We therefore draw on the general principles of the eating disorder guidelines, together with the English (National Institute for Health and Care Excellence, NICE, 2013) [26], American (American Academy of Child & Adolescent Psychiatry, AACAP, 2020; American Family Physician, AFP, 2022) [27,28], Canadian (Canadian clinical practice guidelines 2014; Canadian Paediatric Society, CPS, 2023) [29,30] and Australian (Royal Australian College of General Practitioners, RACGP, 2024) [31] guidelines on anxiety.

Psychoeducation is essential [31], particularly for mild symptoms, alongside interventions with the family and the broader environment (schools and social relationships) to strengthen positive parenting, encourage coping strategies and address stressors [30]. Psychotherapy is the first-line treatment [29,31], with CBT being indicated first for anxiety disorders of mild to moderate intensity (individual, group or online) [26,27,28,29,31]; it is also indicated as first-line treatment for OCD [32]. Acceptance and commitment therapy (ACT) also may be offered [31]. For moderate to severe forms (panic attacks, inability or refusal to attend school, and increased risk of self-harm or suicide attempts), or after partial response to therapy, combining CBT with a selective serotonin reuptake inhibitor (SSRI) improves outcomes and promotes remission [27,28,30,31].

SSRIs (fluoxetine, sertraline, escitalopram, fluvoxamine) are the first-line pharmacological treatment, preferably those with a long half-life (fluoxetine), to limit the risk of discontinuation syndrome [30,31]. Prescribing follows the “start low and go slow” principle [28,31], starting at the lowest dose appropriate for the patient’s age. If the first SSRI fails, a second SSRI is recommended, or, in the event of intolerance, a serotonin–norepinephrine reuptake inhibitor (SNRI: venlafaxine, duloxetine) is indicated [27,28,30,31], with monitoring for activation and increased suicidal ideation, particularly under venlafaxine [28,31]. Treatment continued for 6 to 12 months after remission [28]. Benzodiazepines should be used only for short-term use in the management of acute crisis, never continuously or repeatedly [31].

### 4.2. Proposal for a Clinical Management Strategy for Anxiety Symptoms in Adolescents with Anorexia Nervosa

Based on the results of our scoping review, complemented by the guidelines on anxiety disorders in adolescents, we propose a clinical management strategy covering three clinical situations. This strategy is summarized in Figure 2. Each element of the algorithm is labeled by its source: guidelines on anorexia nervosa, studies on anorexia nervosa identified by the review, guidelines on anxiety disorders in children and adolescents, or the authors’ interpretative synthesis in the absence of any of these. References are given for the first three categories.

#### 4.2.1. Isolated Anxiety Symptoms

Some patients present anxiety symptoms centered on meals, food, body image and weight, with no diagnostic criteria for an anxiety disorder or OCD and no personal history of either. These symptoms are then taken to be isolated, attributable to anorexia nervosa, to undernutrition or to the current clinical course [15,17,62] (see Table 1). The strategy is then to continue nutritional rehabilitation and overall care, with attention to context (relational support before, during and after meals, in a calm setting), together with psychoeducation and biopsychosocial, family and school support. Associated anxiety symptoms should not delay refeeding.

However, if the course is unfavorable (self-harm, anxiety of increasing intensity or blocking nutritional rehabilitation), symptomatic treatment may be added: CBT first, possibly supplemented by low-dose olanzapine, always alongside psychotherapy. This practice rests on a low level of evidence, but is widely used and well accepted by patients [18,33,34,39]. It raises the question of metabolic side effects, especially where binge eating emerges under olanzapine, which would require further research. Complementary or body-oriented approaches (balneotherapy, which may be an alternative to floatation-REST therapy) may be offered as adjuncts.

#### 4.2.2. Comorbid Anxiety Disorders

When DSM-5 [61] diagnostic criteria for an anxiety disorder (generalized anxiety disorder, social anxiety disorder, panic disorder, agoraphobia) or for OCD are met, a genuine comorbid diagnosis is established. In OCD, obsessions and compulsions are ego-dystonic, unlike the ego-syntonic ones found in AN [62]. Management then follows the general treatment framework for anxiety disorders in adolescents, combined with treatment of AN itself. CBT is provided as first-line treatment, and an SSRI is added if the course is not favorable. If anxiety symptoms persist, increase or interfere with nutritional rehabilitation, the guidelines describe the following treatment escalation: continuation of psychotherapy combined with a second-line SSRI, and then with third-line treatment with an SNRI if necessary. Nutritional rehabilitation remains central but treating a comorbid anxiety disorder can promote clinical improvement in AN, although this is a clinical opinion that has not been evaluated in the reported literature.

#### 4.2.3. Uncertain Situations

A family or personal history of anxiety disorders or OCD predating AN points toward comorbidity [61] and indicates that specific treatment should be discussed, which will then follow the management strategy indicated above. When such treatment is not initially initiated, it may be reconsidered whenever the course is unfavorable, or if anxiety persists, increases or interferes with nutritional rehabilitation.

#### 4.2.4. Acute Crisis

When anxiety symptoms are severe and markedly impair functioning in an acute situation, short-term adjunctive anxiolytic treatment with benzodiazepines may be considered, with caution, and neither continuously nor repeatedly [31].

#### 4.2.5. Treatment Initiation and Monitoring

Starting psychotropic medication must be discussed with the patient and, for minors, with their parents, and requires particular vigilance regarding the patients’ physical and nutritional status.

### 4.3. Limitations and Strengths

This work has several limitations. Firstly, the search was structured but possibly not exhaustive, and we cannot exclude the possibility that a guideline published in French or English was missed as there is no specific data base for international guidelines. The second relates to the limitations of the underlying data: most guidelines are based on expert consensus, and no randomized trial has specifically evaluated the pharmacological treatment of anxiety disorders in adolescents with AN. In the absence of specific data, our algorithm is partly based on the transposition of the more general guidelines for the treatment of anxiety in adolescents, whose applicability in AN has not been validated. Moreover, evidence specific to adolescents is scarce. Only two of the eleven guidelines included is specific to children and adolescents; the others address all age groups, and their recommendations derive largely from evidence obtained in adults. Among the 18 publications retrieved, only two reported anxiety outcomes specifically in adolescents, providing very limited evidence. We sought to limit the consequences of this by transposing the therapeutic steps of our algorithm onto the guidelines for the treatment of anxiety disorders in children and adolescents, which are specific to this age group. Their applicability to adolescents with anorexia nervosa has still not been evaluated. Finally, we did not formally appraise the methodological quality of the included studies, as is standard in scoping reviews. Nevertheless, this work is based on diverse and up-to-date sources, international clinical practice guidelines supplemented by the recent literature following PRISMA methodology. The proposed algorithm is based on the current state of knowledge and on still-limited direct evidence. It combines elements derived from clinical practice guidelines and expert consensus. Further research is needed to strengthen the robustness of its evidence base.

## 5. Conclusions

Adolescents with AN frequently present anxiety manifestations whose nature must be established at the initial assessment and re-evaluated regularly: these could be symptoms related to undernutrition, symptoms intrinsic to the eating disorder, or genuine comorbidities. This distinction is based on a history of anxiety disorders, on the course of symptoms during nutritional rehabilitation and on the ego-syntonic or ego-dystonic nature of the obsessions. It guides the therapeutic strategy and helps avoid both delayed treatment of an established disorder and over-prescription in adolescents vulnerable to adverse effects.

Our review confirms that no pharmacological treatment is specifically validated in AN and that the available level of evidence is weak, particularly in adolescents. Priority is given to nutritional restoration and psychotherapy; psychotropic drugs remain reserved for established anxiety comorbidities or highly disabling symptoms and body-oriented approaches are possible adjuncts that require evaluation. In the absence of specific data, our algorithm offers a preliminary framework to support clinical reasoning, articulating the guidelines on AN with those on anxiety disorders in adolescents. Further research is needed to strengthen the robustness of its evidence base. Treating anxiety in adolescents with AN remains a genuine research challenge: randomized trials specifically targeting these comorbidities and evaluating both the efficacy and the safety of treatments in this population are needed.

## Figures and Tables

**Figure 1:**
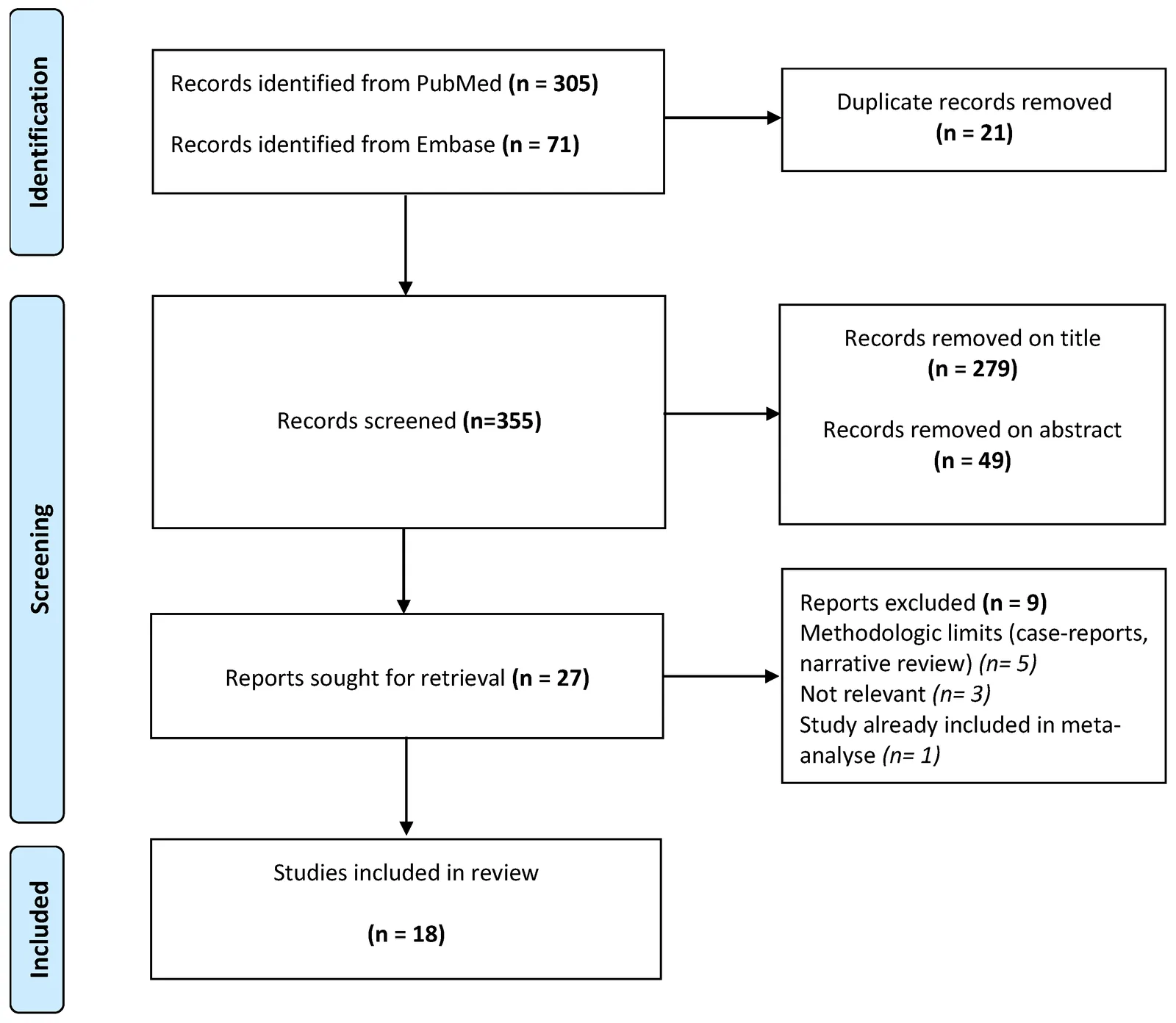
PRISMA flow diagram. Created by the authors, 2026, based on the Preferred Reporting Items for Systematic reviews and Meta-Analyses extension for Scoping Reviews (PRISMA-ScR) [20].

**Figure 2:**
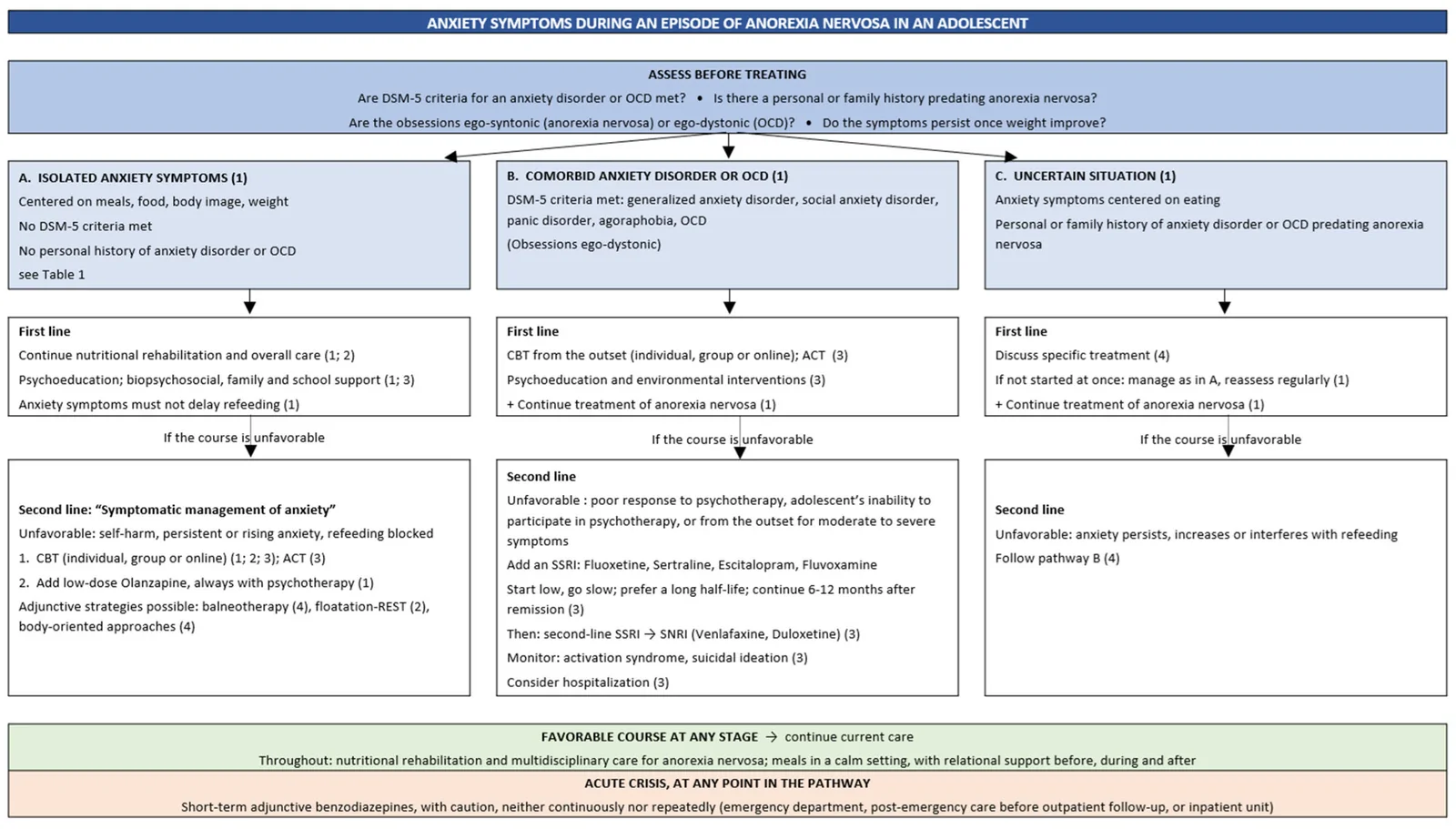
Proposed therapeutic algorithm for anxiety manifestations in adolescents with anorexia nervosa. Created by the authors, 2026, based on [15,16,17,26,27,28,29,30,31,32,33,34,35,37,38,39,40,41,48,49,60,61,62]. Each element is labeled according to its source: (1) guidelines on anorexia nervosa; (2) studies on anorexia nervosa identified by the review; (3) guidelines on anxiety disorders in children and adolescents, transposed as described in the Methods; or (4) the authors consensus-based clinical opinion. Label indicates provenance, not a level of evidence.

**Table 1:** Isolated anxiety symptoms in anorexia nervosa.

**Isolated Anxiety Symptoms Arising from AN, Undernutrition or the Stage of Illness**
- Anxious ruminations focused on body image (including hypochondriacal concerns). - Anxious ruminations focused on calorie control and anticipation of meals. - Obsessive thoughts and behaviors related to food, and eating rituals. - Ego-syntonic obsessions and compulsions. - Exacerbation of anxiety symptoms during nutritional rehabilitation and weight restoration. - Fear of eating in public. - Social withdrawal. - Hyperactivity (suggestive of anxious agitation). - Sleep disturbances (related to hyperactivity and evening anxiety).

Created by the authors, 2026, based on [15,17,62].

## Data Availability

No new data were created or analyzed in this study. Data Sharing is not applicable to this article.

## Supplementary Materials

The following supporting information can be downloaded at https://www.mdpi.com/article/10.3390/nu18213551/s1, Table S1: Guidelines; Table S2: Summary of recent studies investigating treatments for anxiety in anorexia nervosa; Table S3: Search strategy for clinical practice guidelines on anorexia nervosa.

## Abbreviations

The following abbreviations are used in this manuscript:

AACAP	American Academy of Child & Adolescent Psychiatry
ACT	Acceptance and commitment therapy
AFP	American Family Physician
AN	Anorexia nervosa
AWMF	Arbeitsgemeinschaft der Wissenschaftlichen Medizinischen Fachgesellschaften
CBD	Cannabidiol
CBT	Cognitive behavioral therapy
CPS	Canadian Paediatric Society
ED	Eating disorder
floatation-REST	Reduced Environmental Stimulation Therapy
GAD	Generalized anxiety disorder
NICE	National Institute for Health and Care Excellence
NMDA	N-methyl-D-aspartate
OCD	Obsessive–compulsive disorder
PAT-AN	Positive Affect Treatment for Anorexia Nervosa
PRISMA	Preferred Reporting Items for Systematic reviews and Meta-Analyses
PRISMA-ScR	Preferred Reporting Items for Systematic reviews and Meta-Analyses extension for Scoping Reviews
RACGP	Royal Australian College of General Practitioners
rTMS	Repetitive transcranial magnetic stimulation
SNRI	Serotonin–norepinephrine reuptake inhibitor
SSRI	Selective serotonin reuptake inhibitor
tDCS	Transcranial direct current stimulation, tDCS
WFSBP	World Federation of Societies of Biological Psychiatry
