# Supplementary material for: Anxiety Symptoms and Comorbid Anxiety Disorders in Adolescents with Anorexia Nervosa: A Scoping Review and Proposal for a Management Algorithm
Source: Nutrients. 2026 Oct 28;18(21):3551. doi: 10.3390/nu18213551 (PMC13765044; doi:10.3390/nu18213551)
Supplement: Supplementary file 1 [file nutrients-18-03551-s001.zip › nutrients-4510546-supplementary.pdf]

Supplementary Materials

Table S1. Guidelines (in chronological order)

Spanish guidelines: 2009

Based on Ministry of healthcare and consumer affairs. Catalan Agency for Health Technology Assessment and Research, Clinical Practice Guideline for Eating Disorders, 2009

p 26, p 158 and p 202:
"Caution should be exercised when prescribing pharmacological treatment for patients with AN who have associated comorbidities such as obsessive-compulsive disorder (OCD) or depression."

p 28:
"The use of questionnaires adapted and validated in the Spanish population is recommended for the psychopathological assessment of eating disorders. At present, the following instruments for psychopathological assessment of eating disorders are suggested (version selection based on patient's age and other application criteria):
Impulsiveness: BIS-11
Anxiety: STAI, HARS, CETA
Depression: BDI, HAM-D, CDI
Personality: MCMI-III, MACI, TCI-R, IPDE
Obsessiveness: Y-BOCS"

p 52-53:
"Anxiety disorders
Anxiety is so present in eating disorders it is hard to decide if it is a specific clinical component of these disorders or if it is a comorbid condition. Eating disorders present specific phobia clinical pictures (phobia of certain foods, social phobia, etc.) and others such as claustrophobia or unrelated simple phobias. Panic attacks or anxiety crises present a similar situation. In some cases they are linked to eating disorders and in others they are truly comorbid. Studies report very varied anxiety prevalences that range between 10% and 40%, depending on the measurement tools and inclusion criteria used.
Obsessive-compulsive disorder (OCD)
Patients with eating disorders, especially AN, present significant prevalence of obsessive personality traits (See personality disorders). Many of these patients are rigid, strict, organized, responsible, constant, intransigent and intolerant, personality traits that predispose and accompany eating disorders. However, in a considerable number of patients, up to 40% of them diagnosed with AN, obsessive-compulsive disorder that meets comorbidity criteria is present."

p 138:
"Pharmacological treatment
[...]
Although benzodiazepines such as alprazolam and lorazepam are indicated as anti-anxiety drugs no RCTs that address their use for eating disorders have been identified. A similar situation has been encountered with lithium."

p 150:
"In some of the case series and in most of the RCTs, patients with AN gained weight while receiving treatment with olanzapine, achieving healthy weight in many cases. Reported cases and RCTs identified additional benefits of olanzapine, including the reduction of delusional beliefs, improvement of body self-image, decrease of agitation and anxiety prior to meals, improvement of sleep, depressive symptoms and treatment adherence and other symptoms related with eating disorders.

Preliminary evidence supports the use of olanzapine for the treatment of AN, demonstrating that a 2.5 to 15 daily mg dose promotes weight gain and has positive effects on related psychological symptoms. The limitations of the data analysed are: small sample, high dropout rate in the RCT and the open design of the RCT."

p 170:
"Care must be exercised when using medication to treat comorbid conditions such as depression and OCD compulsive symptomatology in patients with AN when these clinical pictures could be resolved through weight gain."

p 241:
"Pharmacological treatment: aimed at patients who require drugs in combination with psychological treatment, especially in cases of BN and BED, or to resolve associated symptomatology such as depression, anxiety or impulsiveness."

French guidelines: 2010 and 2022

Based on Haute autorité de santé (HAS), Anorexia nervosa: management, 2010
HAS, Early-Onset Anorexia Nervosa: French National Diagnostic and Care Protocol, 2022

HAS, Anorexia nervosa: management, 2010:

p 14:
"Objectives of the psychological interventions
[…]
Comorbid disorders, especially depression, anxiety, OCD and addictions, should be taken into account."

p 16:
"Pharmacological treatment
There is no specific medication to treat AN. It should be added that certain drugs, such as antipsychotics and tricyclic antidepressants, should be used cautiously on account of their side effects in severely undernourished subjects (longer QT).
However, antidepressants can be used to treat specific concomitant syndromes (depressive disorders, anxious disorders, OCD) if these symptoms are not improved by the weight gain."

p 19:
"Psychiatric criteria for hospitalisation- Co-morbidity
Any co-occurring psychiatric disturbance where severity warrants hospitalisation:

- Depression
- Substance abuse
- Anxiety
- Psychotic symptoms
- Obsessive-compulsive disorder"

p 30:
"Definition of Anorexia Nervosa
Differential Diagnosis: There may be associated depressive or obsessional symptoms, as well as features of a personality disorder, which may make differentiation difficult and/or require the use of more than one diagnostic code. Somatic causes of weight loss in young patients that must be distinguished include chronic debilitating diseases, brain tumors, and intestinal disorders such as Crohn's disease or a malabsorption syndrome.

HAS, Early-Onset Anorexia Nervosa: French National Diagnostic and Care Protocol, 2022:

p 7:
"Anxiety and depression are common comorbidities, but antidepressants are not recommended in underweight children."

p 18:
"Treatment - Objectives
Psychological follow-up with family therapy recommended as first line treatment in EOAN, and/or CBT for the management of anxiety-depressive comorbidities, OCD, low self-esteem, difficulties in expressing emotions as well as to work on anorexic cognitions and cognitive rigidity"

p 20-21:
"The possible use of anxiolytic drugs (antihistamines, neuroleptics) or antidepressants to relieve symptoms of anxiety and depression or to treat a comorbid major depressive disorder should be administered with caution and with ECG monitoring (pre-therapy and monitoring under treatment) (5,28). The minimum effective dose, the child's compliance and parental agreement should be obtained. In the case of comorbid anxiety or depression, antidepressants such as selective serotonin reuptake inhibitors (SSRIs) are not effective in very undernourished children (23). They should be introduced after sufficient weight has been regained, allowing good tolerance (the risk of iatrogenicity is correlated with the level of undernutrition). SSRIs have not been shown to help prevent relapse of AN after weight restoration (5,23)."

p 21:
"Cognitive and behavioural therapy (CBT) is a validated therapeutic option (24,32) that has been shown to be effective as a first-line treatment for the comorbidities of EOAN in children (anxiety-depressive disorders and OCD)."

Australian and New Zealand recommendations: 2014

Based on Hay P *et al*, Royal Australian and New Zealand College of Psychiatrists (RANZCP) clinical practice guidelines for the treatment of eating disorders, 2014

p 10:
"[…] All forms of anxiety disorder may occur and in one study the most common was social phobia (42%), followed by post- traumatic stress disorder (26%) and generalised anxiety disorder (23%) (Swinbourne *et al.*, 2012). Furthermore, social anxiety is not only related to eating in public but may pre-date the onset of the eating disorder. Rates of comorbidity are similar in men and women (Raevuori *et al.*, 2009). However, clinicians should be aware that depression, obsessional thinking, anxiety and other psychiatric symptoms can represent the reversible effects of starvation on the brain (Keys *et al.*, 1950)."

p 15:
"Pharmacotherapy. Recent systematic reviews of RCTs and meta-analyses of the pharmacological treatment of anorexia nervosa suggest weak evidence for the use of any psychotropic agents (Aigner *et al.*, 2011; Flament *et al.*, 2012; Hay and Claudino, 2012; Kishi *et al.*, 2012). Prescribing for comorbid conditions (e.g. anxiety or mood disorders) is best left until it is clear that such symptoms are not simply secondary to starvation, although low doses of antipsychotics such as olanzapine may be helpful when patients are severely anxious and demonstrate obsessive eating-related ruminations, but more trials are needed (Hay and Claudino, 2012). Caution is required for any psychotropic medication, as physical problems secondary to anorexia nervosa may place individuals at greater risk of adverse side effects."

p 18:
“Specific pharmacological treatments
There is only weak evidence for pharmacological treatment of anorexia nervosa. Low-dose antipsychotics such as olanzapine may be useful in reducing anxiety and obsessive thinking, but results of trials are mixed and such individuals are at greater risk of adverse side effects (EBR I). Exercise caution in prescribing psychotropic medication for severe comorbid conditions/symptoms until it is clear that such symptoms are not secondary to starvation.”

p 19:
“Complications from malnutrition and compensatory behaviours associated with anorexia nervosa include […] psychological complications including depression, anxiety, obsessive compulsive disorder and cognitive impairment (Hatch *et al*., 2010).”

p 22:
“Pharmacotherapy.
There is insufficient evidence to recommend psychotropic medication in adolescents with anorexia nervosa. The use of anxiolytic or antidepressant medications to relieve symptoms should be done with caution.”

p24:
“Summary of recommendations for Anorexia nervosa in adolescents and children
Selective serotonin reuptake inhibitors (SSRIs) are not indicated in the acute or maintenance stages of anorexia nervosa”

p61:
“Table 7: Role and evidence base support for specific treatments in eating disorders.
Antipsychotic medication:
[….]
-Reduced anxiety or ruminations
Antidepressant medication:
[…]
No robust and/or conflicting evidence”

American guidelines (child and adolescent psychiatry): 2015

Based on Lock, La Via, and the American Academy of Child and Adolescent Psychiatry (AACAP) Committee on Quality Issues, Practice Parameter for the Assessment and Treatment of Children and Adolescents With Eating Disorders, 2015

p 414:
“AN and OCD share obsessional preoccupations, such as over eating, food, weight, and shape obsessions, and compulsive behaviors such as restricting and counting calories, overexercising, checking behaviors, and meal-time rituals, making it difficult to differentiate these 2 disorders. When the preoccupations and compulsions emerge at the same time and are focused on food, eating, and weight, the diagnosis, however, is likely to be AN. In addition, comorbid conditions, particularly anxiety disorders, are often present before AN develops and persist after recovery.”

p 418:
“Evaluation of comorbid symptoms including depressive symptoms, anxiety symptoms, obsessional thoughts, self-injurious behavior, and suicidality is also necessary.”

p 420:
“Recommendation 7. The use of medications, including complementary and alternative medications, should be reserved for comorbid conditions and refractory cases [CG]”
“[…] No systematic studies of SSRIs have been conducted in adolescents with AN. More recently, atypical antipsychotics have been examined in small studies and small RCTs in adults because of their potential effects on weight, anxiety, and obsessive thinking.”

p 421:
“For comorbid conditions, guidelines for the specific condition should be followed. When patients are starved, however, lower levels of available serotonin may limit the effectiveness of SSRIs and other antidepressants until weight is at least partially restored. In addition, it is important to note that obsessionality and depressed mood often improve with weight gain alone.”

p 421, Table 1:
“Antidepressants — Treatment targets: Symptoms of obsessionality, anxiety, and depression in AN and BN […] — Recommendations: Useful for comorbid disorders; may be a second-line treatment for adolescent BN”
“Atypical antipsychotics — Treatment targets: Body image distortion, weight-gain fears, and anxiety related to AN — Evidence base: Case series data and 3 pilot RCTs; insufficient evidence to suggest efficacy for use in AN — Recommendations: Useful for comorbid conditions; further study needed to determine efficacy for core symptoms of AN”

German Guideline: 2018

Based on Herpertz S *et al.*, Diagnosis and treatment of eating disorders: German S3-guideline, 2018.

p 116:
“Compared to the group which did not receive olanzapine, the group receiving olanzapine showed significantly better weight gain and a greater decrease in obsessive symptoms.”
“Olanzapine is generally initially used at a low dose of 2.5mg, which can be increased if necessary.”

p 117:
“Antipsychotics serve to reduce stress conditions, the often pronounced urge for movement and the circular thinking around eating disorder- related themes such as eating or shape and weight.”

“From the available case studies, application reports and clinical practice, the following principles can be derived for antipsychotic administration in AN:
1. The required dosage is generally low.
2. The indication is derived from symptoms which accompany the AN. The necessary duration of pharmacological treatment thus depends on the course of these symptoms and not on the eating disorder diagnosis.
3. Antipsychotics with a low range of extrapyramidal side effects are preferred.
4. Compliance is crucial for the utility of such medications. The benefit of the medication must be communicable to the patient.”

"Olanzapine is described as the antipsychotic with the presumably clearest effect on AN, although an effect on the core symptoms of AN have not yet been conclusively demonstrated. However, olanzapine appears to have an effect on the associated symptoms such as compulsions.
[…]
There is limited evidence that obsessive-compulsive symptoms and circular thoughts are favorably influenced by olanzapine (EL 2a)."

"Clomipramine, a tricyclic antidepressant, is at least theoretically an interesting substance for the treatment of AN given its use in the treatment of obsessive-compulsive disorders"

p 119:
"Overview: Evidence on treatment with antidepressants
There is no evidence for a specific efficacy of antidepressants in the treatment of AN if the focus is on weight-related criteria.
Nevertheless, antidepressants are regularly used in anorexia treatment within clinical practice in order to treat concomitant symptoms of AN such as depressive disorders or obsessive- compulsive symptoms. […]
There is no sufficient evidence to justify this practice. Depressive and obsessive-compulsive accompanying symptoms are also caused by the low body weight and can improve through weight gain even without an additional specific therapy (Dowson, 2004; Jordan *et al.*, 2008; Meehan *et al.*, 2006).
Furthermore, the risk of side effects in antidepressant pharmacotherapy is increased in AN due to several specific factors"

p 121:
"Anxiolytics – Benzodiazepines
Benzodiazepines bind to central nervous system inhibiting GABA receptors and exert anxiolytic, muscle-relaxant, anticonvulsive and sleep-promoting effects. Due to the anxiolytic active component, there is at least theoretical reason to assume that administration will influence fears in relation to food intake in AN. However, a high potential for dependence and negative effects on learning processes, which play an important role in the therapy process, strongly limit this treatment approach. With regard to alprazolam, there is one controlled study (Steinglass *et al.*, 2014b) with an extremely short observation period (only two administrations at two mealtimes; N=20). No difference compared to placebo was found regarding amount of food and anxiety."

p 127:
"For patients with other mental disorders (e.g. borderline personality disorder, PTSD), a hierarchization of therapy aims may be necessary (oriented to the degree of risk from the respective symptom). In such cases, the therapeutic approach should contain elements that are aligned to the guidelines for the respective comorbid disorder."

British guidelines: 2017, last revised in 2020

Based on National Institute for Health and Care Excellence (NICE), Eating disorders: recognition and treatment, 2020

p 28:
"Comorbid mental health problems

1.8.12 When deciding which order to treat an eating disorder and a comorbid mental health condition (in parallel, as part of the same treatment plan or one after the other), take the following into account:
• the severity and complexity of the eating disorder and comorbidity
• the person's level of functioning
• the preferences of the person with the eating disorder and (if appropriate) those of their family members or carers.
1.8.13 Refer to the NICE guidelines on specific mental health problems for further guidance on treatment."

p 29:
"Medication risk management
1.8.14 When prescribing medication for people with an eating disorder and comorbid mental or physical health conditions, take into account the impact malnutrition and compensatory behaviours can have on medication effectiveness and the risk of side effects."

p 42:
"Treating an eating disorder in people with a comorbidity
[…]
Why this is important
People with an eating disorder often have physical comorbidities (such as […] or obsessive-compulsive disorder.) However, there is little evidence on which treatments work best for people with an eating disorder and a comorbidity. A modified eating disorder therapy that addresses both conditions may avoid the need for different types of therapy (either in parallel or one after the other). Alternatively, a comorbidity may be severe enough that it needs addressing before treating the eating disorder, or treatment solely for the eating disorder may help with the comorbidity. This is a complex area and likely to depend on the severity of the comorbidity and the eating disorder."

Scottish guidelines: 2022

Based on Scottish Intercollegiate Guidelines Network (SIGN), SIGN 164: Eating disorder, 2022

p 3:
"Common comorbidities and coexisting health issues which have been considered when reviewing the evidence for this guideline are: […] anxiety disorders […] obsessive-compulsive disorder (OCD)"

p 13:
"Few trials have been identified in patients with ED and comorbidities."
[Good practice point] "Alternative choices should be offered when first-line treatments are ineffective or unsuitable for those with moderate to severe comorbid psychiatric disorders. Choice of treatments should take into account therapeutic models that have an established evidence base in the treatment of comorbidities that have been shown to interfere with treatment outcomes in eating disorders (eg post-traumatic stress disorder, personality disorder, substance misuse)."

p 19 (children and young people with anorexia nervosa):

"Evidence on the use of antidepressants (including fluoxetine) in adolescents with AN was of poor quality. Antidepressants had no impact on weight gain and the impact on eating symptoms or psychopathology is unclear."
"However, psychotropic medication, most often olanzapine or fluoxetine, can be prescribed for children and adolescents with eating disorders."

p 20 (children and young people with bulimia nervosa):
"No specific evidence was identified on pharmacological therapies in children and young people under the age of 16. This, however, should not preclude their use for comorbid conditions, such as anxiety disorders, OCD and depression."

p 27 (adults with anorexia nervosa):
"Prescribing for patients with AN always requires consideration of the starved physiology of the patient, their low body weight and abnormal electrolytes due to purging behaviours. Psychotropic and other medication carries risks to the heart and other organs so benefits must always be set against these, and as a result research in this field is scarce."
[Good practice point] "All low-weight patients should be monitored particularly closely when psychotropic drugs are prescribed. A baseline electrocardiogram (ECG) and further monitoring can alert clinicians to avoidable dangers."
"The rationale for using antipsychotic medication for AN is for the reduction of distress and obsessionality."

p 28 (adults with anorexia nervosa):
"Self-administered intranasal oxytocin resulted in a reduction in anxiety levels in response to food and eating for inpatients undergoing nutritional rehabilitation, in a small RCT. Further studies into the benefit of this therapy are required before a recommendation can be made."
[Recommendation] "Olanzapine may be offered to adults with anorexia nervosa to support recovery but should not be offered as the sole treatment."
[Good practice point] "Once the patient is weight restored general guidelines on the management of obsessional symptomatology and treatment for depression, anxiety and other conditions can be followed."
[Good practice point] "Any other medication selected for the symptomatic treatment of anorexia nervosa and comorbid conditions should be carefully monitored and audited if possible. Patients should be entered into available research trials, if they consent."

World Federation of Societies of Biological Psychiatry (WFSBP) guidelines: 2023

Based on Himmerich H et al., WFSBP guidelines update 2023 on the pharmacological treatment of eating disorders, 2023

p 12:
"Serotonin reuptake inhibitors
Fluoxetine
In a randomised double-blind placebocontrolled trial conducted by Attia *et al*. (1998) the augmentation of an inpatient AN program with fluoxetine was investigated in 33 participants. No impact was shown on weight, eating behaviour or psychological state.
A randomised double-blind placebo-controlled trial by Kaye *et al.* (2001) compared the adherence of 39 patients with AN to fluoxetine or placebo over one year. Adherence to fluoxetine was significantly higher, with 10 of 16 patients remaining on fluoxetine as opposed to only three of 19 on placebo. Only drug completers

showed lower relapse and significant prepost improvement in weight, symptoms of depression, anxiety, OCD and EDs.

Walsh *et al.* (2006) conducted another randomised double-blind placebo-controlled trial examining the effect of fluoxetine for relapse prevention. Ninetythree patients were randomised and 53 completed the 1-year study, with a similar proportion of completers in both groups. No difference was found in time-to relapse between the groups, and a drug effect was found only for anxiety symptoms.

With these contradictory results including two negative RCTs and one positive RCT, strong evidence against the use of fluoxetine emerges (LoE: -A), with a strong recommendation against its use (GoR: -1). Nota bene, the evidence against the use of fluoxetine refers to the main AN outcome of the studies (weight gain and AN psychopathology), not depressive or anxious symptoms."

p 14:

"Atypical antipsychotics

Olanzapine

A double-blind randomised placebo-controlled trial by Attia *et al*. (2011) involving two centres tested the effect of olanzapine given in a dose of 2.5-10 mg/day, if tolerated, to outpatients with AN aged 16 or over, with BMI 14–19 for 8 weeks. The researchers had been in contact with 603 patients with AN, of whom 87 were eligible and agreed to a telephone screening interview. However, about half of the patients did not attend the in-person evaluation, others were not interested in the study or were lost due to other reasons. Therefore, only 23 were randomised of which 17 patients (74%) completed the study revealing a significant drug effect on weight gain but not on psychological symptoms including depression, anxiety and ED symptoms.

These findings were replicated in a larger multicentre double-blind randomised placebo-controlled trial of adult outpatients with AN (Attia *et al.* 2019). One hundred fifty-two participants from five centres were randomised to receive placebo or 2.5-10 mg/day of olanzapine. The completion rate of the study was 55% (n=83), and intention-to-treat analysis showed a significantly greater BMI increase in the olanzapine group. No group differences were observed for the psychological symptoms.

[…]

In a double-blind, placebo-controlled trial Bissada *et al*. (2008) randomised 34 patients with AN (mean age: 26.8 years) to receive 2.5–10 mg/day of olanzapine or placebo over 10 weeks in a day-care treatment program. The olanzapine group was significantly superior over the control group concerning rate of weight gain, earlier achievement of the target BMI and reduction of obsessive (but not compulsive) symptoms as measured by the Y-BOCS. No effect was observed for depression or anxiety symptoms.

Kafantaris *et al*. (2011) conducted a double-blind randomised placebo-controlled trial in 20 adolescents with AN-R up to the age of 21, with 2.5–10 mg/day of olanzapine or placebo. Both groups had similar weight gain and resting energy expenditure and no differences in psychological symptoms. A trend for increasing fasting glucose and insulin levels was found only in the olanzapine group at week 10.

Considering the evidence from five RCTs, four of which showed a significant effect on weight gain (Brambilla *et al*. 2007; Bissada *et al*. 2008; Attia *et al*. 2011, 2019), we conclude a strong level of evidence for olanzapine in AN (LoE: A). However, the reluctance of patients to take olanzapine (Attia et al. 2011), low adherence rates (Attia *et al.* 2019), moderate acceptably and reports of either hyper- or hypoglycaemia lead to a limited recommendation for olanzapine (GoE: 2) in adult patients.

Regarding adolescents, a protocol for a randomised double-blind placebo-controlled trial for the evaluation of efficacy and safety of olanzapine as an adjunctive treatment for AN in adolescent females was published in 2008 (Spettigue *et al*. 2008). The study protocol was modified, and the study was reported as an open-label study (Spettigue *et al.* 2018) which examined the effectiveness and safety of olanzapine in 32 adolescents with AN: 14 in the intervention group and 18 in the comparison group of whom eight switched from no adjunctive medication to olanzapine (Spettigue *et al.* 2018). A higher rate of weight gain was demonstrated in the olanzapine group, with no advantage in psychological symptoms. There were more abnormal chemistry results

in the intervention group including elevated liver enzymes, cholesterol and asymptomatic prolactin levels. However, no elevated glucose levels or HBA1C were recorded.
Ayyıldız *et al*. (2016) reported a case of a 17-year old male inpatient with AN-B/P and BMI 11.9, who developed neuroleptic malignant syndrome after two days of treatment with olanzapine 5 mg/day. The illness presented with fever, muscle rigidity, and autonomic instability, including a second episode after the discontinuation of the medication.
An open trial by Leggero *et al.* (2010) evaluated the effect of olanzapine (mean dose 4.13 mg/day) on 13 girls with AN-R aged 9–16 years. Improvements were found in BMI, ED symptoms, anxiety, depression, and hyperactivity. Authors noted the improvement in hyperactivity distinguished responders from nonresponders."

p 15
"Aripiprazole. Trunko *et al.* (2011) reported five cases of AN treated with aripiprazole 5–10 mg/day, and described weight increase, mood elevation, and a reduction in eating-specific anxiety, and decreased rigidity. Frank (2016) reported four adolescent AN cases treated with aripiprazole: three 12 year-olds and one 17 years-old, achieving weight gain and stabilisation as well as general psychosocial improvement. In one case drug-induced neutropenia was observed but the drug was maintained under monitoring due to the beneficial effect on eating-anxiety and to the patient's request.
[…]
Another retrospective case-controlled study was conducted by Marzola *et al*. (2015) comparing three groups of adults patient treated with: (1) SSRI only, (2) SSRI and olanzapine, or (3) SSRI and aripiprazole. All groups improved in depressive, anxiety, and ED symptoms as well as weight gain. A greater reduction in ED rituals and pre-occupations was found in the aripiprazole group compared to olanzapine augmentation and SSRI only. An additional finding was a decrease in purging in the aripiprazole group vs. the olanzapine group.
Overall, the evidence for aripiprazole (in adolescents and adults) comprises two retrospective case control study and three case series, thus limited evidence (LoE: C1 and weak recommendation for its use (GoR: 3)."

p 16:
"Antiepileptics and mood stabilisers
Lithium
A single placebo-controlled double-blind trial with Lithium was conducted by Gross *et al.* (1981) on
16 patients with AN aged 12–32. There was increased weight gain in weeks 3–4 of the trial but not in weeks 1–2. Denial of illness and selective appetite were the only psychological assessments that significantly differed between groups with no differences in depression, anxiety, or obsessive symptoms. This study suggests limited evidence for Lithium (LoE: B), and weak recommendation, because of considering significant side effects and required monitoring, and no further evidence accumulated since this study (GoR: 3)."

p 18:
"Testosterone
Kimball *et al.* (2019) reported an RCT in 90 female patients with AN testing 300 lg transdermal testosterone daily or a placebo patch for 24 weeks. Testosterone was associated with less weight gain and did not lead to improvements in depression, anxiety, or disordered eating symptoms-compared with placebo in women with AN. Thus, there is limited evidence (LoE: B) and a limited recommendation against the use of transdermal testosterone (GoR: -2)."

p 18:
“Nutritional supplements
Zinc
Two randomised double-blind placebo-controlled trials were conducted to investigate the effect of zinc supplantation in patients with AN. Katz *et al.* (1987) studied 15 adolescents and while no effect on weight gain was demonstrated, depression and anxiety symptoms improved in the zinc group. Birmingham *et al*. (1994) studied a mixed population (N=35) with an age-range of 12–25 years, with a significant increase in weight gain for the zinc group. These conflicting results from limited studies which were not further pursued represent insufficient evidence (LoE: D) to draw a recommendation (GoR:4).

Polyunsaturated fatty acids (PUFA)
A pilot study by Manos *et al.* (2018) compared the outcomes with four daily doses of omega-3 PUFA supplementation or placebo in a double-blind, placebo-controlled randomised trial of adolescent females with AN (N=24). No benefit was shown for weight or psychological symptoms. In conclusion, this study provides limited evidence against PUFA (LoE: -B), and a limited recommendation against its use (GoR: -2).

Tyrosine
Hart *et al*. (2021) reported two cases in which amino-acid tyrosine was given for 12 weeks. One of the two gained weight and improves depressive and OC symptoms. This is a non-analytic report
with low evidence (LoE: C2), and a weak recommendation (GoR: 3).”

p 18:
“Other medications
Alprazolam
Steinglass *et al.* (2014) performed a double-blind placebo-controlled crossover study to examine the effect of benzodiazepine treatment (alprazolam 0.75 mg) on meal anxiety and caloric intake at meal. No differences emerged between the drug and placebo condition, although alprazolam resulted in greater fatigue. As this is a single meal study, there is not sufficient data (LoE: D) on this intervention (GoR: 4).”

p 19:
“D-cycloserine. Steinglass *et al.* (2007) conducted an RCT in 14 patients to compare the adjunctive administration of d-cycloserin before four meal-exposure sessions. Caloric intake was not different across interventions. A similar trial by Levinson *et al*. (2015) where c-cycloserine or placebo were given to 36 patients before three exposure sessions over 2 weeks and with 1-month follow-up, and resulted in an increase in BMI compared to the placebo group, although mealtime anxiety was unaffected. These limited data are insufficient (LoE: D) for any further recommendation (LoR: 4).”

p 19:
“Ketamine. Scolnick et al. (2020) reported a case of a 29 year old female with chronic AN who attained a 6-month stable remission from symptoms and weight restoration with ketogenic diet and ketamine. Several case studies have been reported in patients with depression and AN. Dechant et al. (2020) reported on patient with AN and depression who experienced a reduction in depression and suicidality. In a case series published by Mills *et al.* (1998), nine of 15 responded to treatment, with reductions in depression. And improvement in AN behaviour and psychopathology.
Four cases published by (Schwartz *et al.* 2021) showed improvements in depression, anxiety, and eating disorder psychopathology. These case reports show limited evidence (LoE: C2) and weak recommendation for ketamine in combination with a ketogenic diet (GoR: 3).

p 52:
Whether a study was successful or not was decided with reference to the main outcome and improvement of diagnostic criteria. Other important outcomes for patients were therefore potentially neglected. For example, in the study of Walsh *et al*. (2006) no difference was found in time-to-relapse between the fluoxetine and the placebo group. However, a drug effect was found for anxiety symptoms. For an individual patient, a reduction of anxiety might be an important outcome. Indeed, most patients with AN would find medication useful if it helped reduce anxiety or sleep problems (Tyrrell-Bunge *et al.* 2018). Indeed, Patient Reported Outcome Measures (PROMs) and Patient Reported Experience Measures (PREMs) are increasingly perceived as clinically important as they assess the efficacy, safety, and experience of care from a patient perspective (Black et al. 2014). Specific PROMs and PREMS for EDs have not been developed yet. Therefore, future studies and guidelines might be able to take the patients' perspective into account."

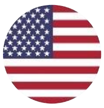

American guidelines: 2023

Based on American Psychiatric Association (APA), The APA practice guideline for the treatment of patients with eating disorders, 2023

p 18:
"The relationship between eating disorders and psychiatric symptoms is complex; careful clinical assessment is needed to discern whether symptoms of depression, anxiety, or obsessionality reflect an independent co-occurring disorder or have developed as a result of the eating disorder"

p 36:
"Psychological considerations during nutritional rehabilitation
(…) it is important to warn patients about the following aspects of early recovery: as they start to recover and feel their bodies getting larger, especially as they approach numbers on the scale that represent phobic weights, individuals may experience a resurgence of anxious and depressive symptoms, irritability, and sometimes suicidal thoughts. These mood symptoms, non-food-related obsessional thoughts, and compulsive behaviors, although often not eradicated, will usually decrease over several months following weight restoration if weight is maintained and restrictive eating, binge eating, or purging behaviors do not recur. If mood symptoms and non-food-related obsessions and compulsions persist, further assessment should occur to identify co-occurring disorders and implement additional treatment for any such disorders, as appropriate."

p 37:
"The limited empirical data do not show any advantages of selective serotonin reuptake inhibitors (SSRIs) in terms of weight gain however, these medications are commonly used […] are relatively well tolerated, and may be considered for those with persistent depressive, anxiety, or obsessive-compulsive symptoms. If antidepressants are considered for co-occurring disorders in adolescents and emerging adults, clinicians should attend to the boxed warnings relating to antidepressants and discuss the potential benefits and risks of antidepressant treatment with patients and families (U.S. Food and Drug Administration 2018).
[…]
Medication such as OLANZAPINE may be useful in selected patients to assist with weight gain; however, potential adverse effects (e.g., glucose dyscontrol, metabolic syndrome, akathisia, extrapyramidal effects) need to be considered (Attia *et al*. 2019).
Despite the high levels of anxiety in some patients related to eating, use of a BENZODIAZEPINE as an anxiolytic agent does not appear to be beneficial (Steinglass *et al*. 2014) and carries a risk of misuse."

p 38: “Weight maintenance/stabilization:
The available evidence does not suggest a specific benefit for use of SSRIs in addition to CBT in reducing relapse risk in patients whose weight has been restored (Walsh *et al*. 2006), although these medications may be used to treat co-occurring disorders during the weight maintenance phase of treatment.”

Brazilian guidelines: 2026

Based on Appolinario *et al*., Brazilian Psychiatric Association guidelines for the treatment of eating disorders, 2026 (accepted pre-proof)

p 9:
“Optimal outcomes arise when nutritional therapy complements effective psychotherapies, such as cognitive-behavioral therapy for EDs (CBT-ED) and family-based treatment (FBT), fostering improvements in mood, anxiety, (LOE 1) (12) and supporting long-term recovery (LOE 2) (28).”

p 10:
“Drug treatments in AN aimed at improving weight gain and reducing the core preoccupations and behaviors of the disorder, as well as comorbid mood or obsessive-compulsive symptoms, either in the acute phase or as a form of relapse prevention after weight restoration.”

p 11:
“Systematic reviews and meta-analyses in AN do not support the use of antidepressants (tricyclics, SSRIs) neither to treat ED-specific psychopathology, nor to obtain weight gain in the acute or maintenance phases (34)(35) (LOE=1). In recent decades, research on second generation antipsychotics has increased due to their effects on a broad range of symptoms present in AN (anxiety, depressive, obsessive-compulsive, and psychotic-like symptoms, as well as being underweight).”
“While olanzapine is not approved for the treatment of AN, it may be considered as an adjunct to psychological therapy in cases of insufficient weight recovery (for example due to cognitive rigidity or psychotic-like symptoms). Close monitoring of glucose and lipid metabolism is recommended if olanzapine is prescribed.”

p 23, Table 1 (Summary of Recommendations for the Treatment of Anorexia Nervosa), Pharmacological treatment (LOE 1):
“Pharmacological treatment should not be used as monotherapy for anorexia nervosa. Antidepressants are not recommended for weight restoration or improvement of core eating-disorder psychopathology. Olanzapine may be considered as an adjunctive treatment in selected severe cases because of its small effect on weight gain, with appropriate metabolic monitoring.”

Table S2. Summary of recent studies investigating treatments for anxiety in anorexia nervosa (when anxiety was a secondary study outcome, the reported sample sizes and results are those corresponding to that outcome)

Title	Year	First author	Study type	Number of studies<br>Number of participants (N)	Country	Population<br>Mean age<br>Gender	Diagnoses	Intervention	Outcomes assessed	Main findings<br>Effect size
Noninvasive Brain Stimulation as a Novel Treatment Approach for Anorexia Nervosa: A Meta-analysis and Systematic Review of Literature	2026	Ramana-than	Meta-analysis	3 studies<br><br>N = 82	UK	Adults<br>1) Age: 30<br>F: 100%<br>2) Age :27<br>F: 100%<br>3) Age: 28<br>F: 100%	AN	rTMS (real vs sham)	DASS-21	Significant decrease of anxiety scores after rTMS<br>SMD = −0.461<br>Z = 2.03<br>95% CI: [−0.892; −0.024]<br>p = 0.040, I^2 = 0 %
Efficacy of transcranial magnetic stimulation in anorexia nervosa: a systematic review and meta-analysis	2025	Bahadori	Meta-analysis	3 studies<br><br>N = 57	UK, Canada	Adults<br>1) Age: 30<br>Range: [21-56]<br>F: 100%<br>2) Age: 31<br>F: 100%<br>3) Age: 33<br>F: 100%	AN	rTMS<br>(real vs sham for one study)<br>dTMS	DASS-21<br>BAI	Significant post-intervention amelioration in anxiety<br>(Effect size: NR)
Cognitive-behavioural therapy (CBT) for outpatients with anorexia nervosa: a systematic review and meta-analysis of clinical effectiveness	2025	Duggan	Meta-analysis	3 studies<br><br>N = 58	Australia, China, Japan	Adolescents and adults<br>1) Age: NR<br>F:100%<br>2) Age: 17 (median)<br>F: 94%<br>3) Age: 30<br>F: 100%	AN	CBT	"Most commonly used measures" : PHQ-9, GAD-7, CIA	Medium significant post-CBT decrease in anxiety<br>g = −0.63<br>95% CI: [−1.48; – 0.22]<br>Z = −3.20<br>p < .001

Psychopharmacology of eating disorders: Systematic review and meta-analysis of randomized controlled trials	2023	Fornaro	Meta-analysis	3 studies<br><br>N = 209	Canada, US	Adults<br>1) Age: 28<br>F: 96 %<br>2) Age: 29<br>F: 96 %<br>3) Age: 27<br>F: 100 %	AN	Olanzapine vs placebo	ZAI<br>PAI	Non-significant trend on anxiety symptoms<br>Hedges'g = 0.263<br>95%CI: [-0.007;0.532]<br>p = 0.056; I^2 = 0 %
				3 studies<br><br>N = 159	Canada, US	Adults<br>1) Age:26<br>F: 100%<br>2) Age:22<br>F:100%<br>3) Age: 24<br>F: 100%	AN	Fluoxetine vs placebo	Y-BOCS<br>Y-BCEDS	Significant improve of obsessive-compulsive symptomatology<br>Hedges'g = 0.416<br>95% CI: [0.106-0.727]<br>p = 0.009; I^2 = 0%
Omega-3 as an adjuvant in the treatment eating and psychological symptoms in patients with anorexia nervosa: a systematic review and meta-analyses	2023	Candido	Meta-analysis	2 studies<br><br>N = 27	US	Adolescents and adults<br>1) Age: 15<br>F: 100%<br>2) Age: 25<br>F: NR	AN	Omega-3 supplementa-tion	BAIT<br>STAI	No evidence of effect on anxiety symptoms<br>SMD: 0.79<br>95% CI: [−0.08; 1.66]<br>p = 0.08; I^2 = 3%
The pharmacological treatment of anxiety in people with eating disorders: A systematic review	2025	Morris	Systematic review	8 studies on antidepres-sants<br><br>N = 213	Italy, US, Japan	Adults<br>Age: NR<br>F: NR	AN<br><br>(30% AN and 70% BN for Milnacipran)	Fluoxetine (5 studies)<br>Sertraline (1)<br>Citalopram (1)<br>Milnacipran (1)	HARS<br>SCL-90<br>SCL-58<br>STAI<br>BAI	Evidence for Fluoxetine to reduce anxiety, mostly demonstrated when BMI > 19
				12 studies on antipsychotics<br><br>N = 291 including<br>N = 41 adolescents,	US, Canada, Australia, Italy	Adolescents and adults<br>Age: NR<br>F: NR	AN	Olanzapine (6 studies)<br>Quetiapine (3)<br>Aripiprazole (2)<br>Risperidone (1)	BAI<br>STAI<br>MASQ<br>MASC<br>HARS	Evidence for Olanzapine and Quetiapine to reduce anxiety<br>In studies on Olanzapine among adolescents:<br>- Significant decrease in anxiety but no difference with the care as usual

				in Olanzapine studies						group in an open-label study (N=32)<br>- Reduction of pre-meal anxiety and anxiety around eating in case reports
				1 study on anxiolytics<br><br>N = 20	US	Adults<br>Age: 26<br>F: 100%	AN	Alprazolam	STAI	No significant reduction of anxiety and no difference compared to placebo in a randomised double- blind placebo cross-over study t_{15}=-0.526; p=0.61
				5 studies on psychedelics and cannabinoids<br>N = 29	US<br><br><br>Israël	Adults<br>Age: NR<br>F: NR	AN	Ketamine (3 studies)<br>Psilocybin (1)<br>Δ9-THC (1)	STAI<br>PASTAS<br>HARS<br>GAD-7	Preliminary evidence for the use of Ketamine and Psilocybin<br>No significant change with THC
Psilocybin in the treatment of eating disorders: a systematic review of the literature and registered clinical trials	2025	Bevione	Systematic review	1 study<br><br>N = 10	US	Adults<br>Age: 28<br>F: 100%	AN	Single dose of psilocybin with psychological support	STAI<br>PASTAS	Significant decrease in trait anxiety and in the trait body image anxiety at 1-month follow-up<br>STAI: p = 0.036, *d* = 0.78<br>PASTAS: p = 0.04, *d* = 0.76
Effects of microbiota-based interventions on depression and anxiety in children and adolescents—A systematic review	2025	Hu	Systematic review	1 study<br><br>N = 24	US	Adolescents<br>Age: 15 (PUFAs group) and 14 (control group)<br>F: 100%	AN	12-week Omega-3 Poly-Unsaturated Fatty Acids (PUFAs) supplementation	BAI-Trait	No significant difference on anxiety traits in PUFAs group compared to the placebo group<br>p = 0.55
Harnessing Immersive Virtual Reality: A Comprehensive Scoping Review of its	2024	Di Natale	Scoping review	3 studies<br><br>N = 147	UK, Italy	Adolescents and adults<br>1) Age: 22 Range: [14; 50]	AN	Immersive Virtual Reality	DASS-21	Lower levels of anxiety toward virtual foods in the positive mood condition, compared to the baseline

Applications in Assessing, Understanding, and Treating Eating Disorders						F: 96.55%<br>Non-F or M: 2.07%				condition with medium effect size in a controlled trial<br>F(2,141) = 4.36<br>p = 0.015
					Spain	2) Age: 14<br>F: 100%<br>3) Age: 15<br>F: 100%			PASTAS	Reduction in body-related anxiety after virtual body exposure in two single-case studies
Neuromodulation in the treatment of anorexia nervosa – a literature review	2025	Metelska	Narrative review	2 studies<br><br>N = 6	France	Adults<br>1) Age: 21<br>F: 100%	AN	ECT	HADS	Slight reduction in anxiety in a single case study after ECT
					UK	2) Age: NR<br>F: 100%		rTMS	DASS-21	Improvement in anxiety after rTMS in a 5-case series
Pharmacotherapeutic strategies for the treatment of anorexia nervosa - novel targets to break a vicious cycle	2024	Kirchberg	Narrative review	1 study on anxiolytics<br><br>N = 20	US	Adults<br>Age: 26<br>F: 100%	AN	Alprazolam	STAI	No significant reduction of anxiety and no difference compared to placebo in a randomised double- blind placebo cross-over study<br>p = 0.61
				3 studies<br><br>N = 5	Switzer-land, Germany	Adolescents and adults<br>1) Age: NR<br>F: 100%<br>2) Age: 15<br>F: 100%<br>3) Age: 16<br>F: 100%	AN	Metreleptin	Visual analogue scales	Decrease in food-related thoughts
Olanzapine use for the treatment of adolescents with anorexia nervosa – reflecting on research and clinical practice	2024	Spettigue	Narrative review	2 studies<br><br>N = 22	US	Adolescents and adults<br>1) Age: NR (adolescents)<br>F: NR<br>2) Age: 21<br>F: NR	AN	Olanzapine	STAI	Decrease of reported anxiety around eating in a study of 5 cases<br>Significant reduction in anxiety in an open-label study (17 participants)<br>p = 0.014

Microbiota in anorexia nervosa: potential for treatment	2023	Landini	Narrative review	No clinical studies on the treatment of anxiety in AN using microbiota-targeted interventions	NA	NA	AN	Faecal microbiota transplanta-tion<br>Probiotics and prebiotics supplementa-tion	NA	Improvements of some AN symptoms after FMT in single-case studies<br>Reduction in stress and anxiety symptoms with probiotic supplementation
A randomized, controlled pilot study of positive affect treatment adapted for anorexia nervosa	2024	Haynos	Randomized controlled trial	N = 20	US	Adults<br>Age: 37<br>Range: [19-70]<br>F: 95%	AN	Positive Affect Treatment (PAT): cognitive, behavioral, and mindfulness interventions (control group: “waitlist” = care as usual)	STAI	Improvements in anxiety symptoms in PAT-group after treatment and at 3-month follow-up:<br>*d* = 0.79, 0.80<br>Smaller changes for participants who opted to receive PAT after waitlist (N=4): *d* = 0.20
Virtual food exposure with positive mood induction or social support to reduce food anxiety in anorexia nervosa: A feasibility study	2024	Natali	Randomized study	N = 145	UK, Italy	Adolescents and adults<br>Age: 22<br>Range: [14; 50]<br>F: 96.55%<br>Non-F or M: 2.07%	AN	Virtual reality:<br>1) Food exposure (baseline condition)<br>2) + positive mood induction<br>3) + social support	DASS-21	Lower levels of anxiety toward virtual foods in the positive mood condition, compared to the baseline condition with medium effect size<br>F(2,141) = 4.36<br>p = 0.015
The impact of floatation therapy on body image and anxiety in anorexia nervosa: a	2023	Choquette	Randomized controlled trial	N = 68	US	Adolescents and adults<br>Age:<br>- floatation group: 20	AN	Reduced environmental stimulation therapy via floatation	STAI	Significant reduction in state anxiety from pre- to post-session<br>Δm = −15.75;<br>95% CI: [−17.95; −13.56]<br>p < 0.0001, Cohen’s *d* = 1.52

randomised clinical efficacy trial						- care as usual: 19<br>F: 100%				No significant session by treatment interactions for longitudinal outcome of trait anxiety ($p > 0.05$)<br>(no comparison with control group for secondary outcomes of the study)
Associations between nutritional intake, stress and hunger biomarkers, and anxiety and depression during the treatment of anorexia nervosa in adolescents and young adults	2023	Lin	Observational study	N = 50	US	Adolescents and young adults<br>Age: 16 (median)<br>Range: [11-26]<br>F: 100%	AN	Reported nutritional intake and hunger/stress hormones (cortisol, leptin, adiponectin)	STAI	Significant improvements in state/trait anxiety over six months<br>STAI: −9.0 [IQR=25.0]<br>$p < 0.01$
Reducing State and Trait Anxiety Through Art Therapy in Adolescents with Eating Disorders: Results from a Pilot Repeated-Measures Study	2025	Monaco	Pre-post study	N = 19	Italy	Adolescents<br>Age: 18<br>Range: [14-23]<br>F: 100%	68% AN<br>32% BN	Textile-based art therapy sessions	STAI	Significant state anxiety decrease immediately after sessions<br>$p = 0.002$

AN: Anorexia Nervosa; **BAI**: Beck Anxiety Inventory; **BN**: Bulimia Nervosa; **CI**: Confidence Interval; **CIA**: Clinical Impairment Assessment; **CBT**: Cognitive-Behavioural Therapy; **DASS-21**: Depression Anxiety Stress Scale; **Δ9-THC**: delta-9-tetrahydrocannabinol; **FMT**: Faecal Microbiota Transplantation; **GAD-7**: Generalised Anxiety Disorder Questionnaire; **dTMS**: deep Transcranial Magnetic Stimulation; **F**: female; **HADS** : Hospital Anxiety and Depression Scale; **HARS** =Hamilton Anxiety Rating Scale; **M**: Male; **MASC**: Multidimensional Anxiety Scale for Children; **MASQ**: Mood and Anxiety Symptom Questionnaire; **NA**: Not Applicable; **NR**: Not Reported; **PAI**: Personality Assessment Inventory; **PASTAS**: Physical Appearance State and Trait Anxiety Scale; **PAT**: Positive Affect Treatment; **PHQ-9**: Patient Health Questionnaire-9; **PUFAs**: Poly-Unsaturated Fatty Acids; **rTMS**: repetitive Transcranial Magnetic Stimulation; **SCL**: Symptom Checklist; **SMD**: Standardised Mean Difference; **STAI**: State-Trait Anxiety Inventory; **Y-BCEDS**: Yale-Brown Cornell Eating Disorder Scale; **Y-BOCS**: Yale-Brown Obsessive Compulsive Scale; **ZAI**: Zung Anxiety Inventory

Table S3. Search strategy for clinical practice guidelines on anorexia nervosa
Presentation adapted from the French National Authority for Health methodological guide (HAS, 2012).

Date of search	15 October 2026 (PubMed, agency and society websites)
Period	No date restriction; the most recent version of each guideline was retained
Languages	French, English (full text)
Bibliographic database	PubMed (MEDLINE)
PubMed equation	("Feeding and Eating Disorders"[Mesh:NoExp] OR "Anorexia Nervosa"[Mesh] OR "eating disorder*"[tiab] OR "anorexia nervosa"[tiab]) AND (guideline[pt] OR practice guideline[pt] OR guideline*[ti] OR recommendation*[ti] OR consensus[ti])
Websites of agencies and learned societies (search terms "anorexia nervosa", "eating disorders")	France: Haute Autorité de Santé (HAS), Fédération Française Anorexie Boulimie (FFAB). United Kingdom: National Institute for Health and Care Excellence (NICE), Scottish Intercollegiate Guidelines Network (SIGN), Royal College of Psychiatrists. United States: American Psychiatric Association (APA), American Academy of Child and Adolescent Psychiatry (AACAP), American Academy of Pediatrics (AAP), Academy for Eating Disorders (AED), Society for Adolescent Health and Medicine (SAHM). Canada: Canadian Paediatric Society (CPS). Australia and New Zealand: Royal Australian and New Zealand College of Psychiatrists (RANZCP), National Health and Medical Research Council (NHMRC). Germany: Arbeitsgemeinschaft der Wissenschaftlichen Medizinischen Fachgesellschaften (AWMF). Spain: GuíaSalud. Denmark: Danish Health Authority. Ireland: Health Service Executive (HSE). International: World Federation of Societies of Biological Psychiatry (WFSBP), European Society for Child and Adolescent Psychiatry (ESCAP).
Other sources	Reference lists of included guidelines and of two syntheses of eating-disorder guidelines (Hilbert et al., 2017; Bohon et al., 2025); completeness check against the authors' initial identification, including a generative AI search (Copilot), not reproducible
Inclusion criteria	(a) Comprehensive guideline on the specialist management of eating disorders including AN, developed by or for psychiatric or multidisciplinary eating-disorder services; (b) full text in French or English; (c) issued by a government agency or a national or international learned society; (d) most recent version
Exclusion criteria	Guidelines intended exclusively for primary care or community paediatric practice (recognition, initial assessment, referral); guidelines restricted to a single aspect of care (screening, dietetic, somatic, emergency management, transitions of care) or to a specific population (e.g., higher weight, athletes); consensus documents without institutional endorsement; superseded versions. Exception: WFSBP guidelines on pharmacological treatment, retained because they address the central topic of this review

Records identified	PubMed: 342. Websites of agencies and learned societies: 14 guideline documents. Other sources (reference lists, completeness check): 0 additional. Duplicates removed: 4. Records screened: 352
Excluded on title/abstract (n = 337 records), with reasons	Not on the management of eating disorders (n = 136). Not a guideline, position or consensus document issued for clinical practice — research, reviews, commentaries, news items, implementation or Delphi studies, single-centre protocols (n = 158). Superseded by a more recent version (n = 16): APA (1993, 2000, 2006); Society for Adolescent Medicine (1995) and SAHM (2015) position papers; American Dietetic Association (2001, 2006); Finnish guidelines for children and adolescents (2002, 2003, 2009); AAP (2003); RANZCP (2004); NICE CG9 (2004) and NG69 (2017 version); Canadian Paediatric Society guidance on family-based treatment (2010); WFSBP (2011). Restricted to one aspect of care or to a specific population (n = 16 records, 15 documents): French paediatric nutritional guidelines (2002); American Dietetic Association (2011) and Academy of Nutrition and Dietetics (2020); Brazilian Medical Association guideline on zinc (2013); ACOG gynecologic care (2018); AIS/NEDC position statement on athletes (2020); Canadian consensus on virtual care (2021); SAHM refeeding hypophosphatemia and medical management (2022); guideline for people with higher weight (2022); US Preventive Services Task Force screening statement (2022); Royal College of Psychiatrists MEED (2022, updated 2025); UK experts' consensus on programme-led interventions (2023); Canadian guideline on transitions of care (2025); FIGIJ/NASPAG advocacy statement (2025). Not in French or English (n = 9 records, 6 documents): Swedish consensus (1993); Italian consensus on levels of care (2010); Finnish Current Care guideline (2015); Danish Health Authority (2016); SENPE consensus documents (2018, 2024). Intended for paediatricians in primary care, as stated in the title (n = 2): Chilean Society of Pediatrics (2020); European Academy of Paediatrics (2022).
Full texts assessed	15 (11 included, 4 excluded)
Excluded after full-text assessment (n = 4), with reasons	Intended exclusively for paediatricians or primary care (n = 2): AAP clinical report (2021); Canadian Paediatric Society position statement (2024). Restricted to one aspect of care (n = 1): HSE Ireland, management of the paediatric patient in paediatric units (2024). Not issued by a government agency or learned society (n = 1): Canadian practice guidelines for children and adolescents (Couturier et al., 2020).
Guidelines included (n = 11)	Spanish (GuíaSalud, 2009); French (HAS, 2010); Australian and New Zealand (RANZCP, 2014); American child and adolescent psychiatry (AACAP, 2015); German (AWMF S3, 2018); British (NICE NG69, 2017, revised 2020); French early-onset AN (HAS, 2022); Scottish (SIGN 164, 2022); WFSBP (2023); American psychiatry (APA, 2023); Brazilian Psychiatric Association (2026)
